# Microenvironmental Hypoxia regulates FLT3 expression and biology in AML

**DOI:** 10.1038/srep17550

**Published:** 2015-11-30

**Authors:** Silvia Sironi, Michaela Wagner, Alexander Kuett, Heidrun Drolle, Harald Polzer, Karsten Spiekermann, Christina Rieger, Michael Fiegl

**Affiliations:** 1 Department of Internal Medicine III, Klinikum der Universität München, Munich, Germany; 2 Clinical Cooperation Group Leukemia, Helmholtz Zentrum München, German Research Center for Environmental Health, Munich, Germany; 3 German Cancer Consortium (DKTK), Heidelberg, Germany; 4 German Cancer Research Center (DKFZ), Heidelberg, Germany

## Abstract

Fms-like tyrosine kinase 3 (FLT3) is a receptor tyrosine kinase constitutively expressed by acute myeloid leukaemia (AML) blasts. In addition, 25% of AML patients harbour a FLT3-ITD mutation, associated with inferior outcome due to increased relapse rate. Relapse might be propagated by interactions between AML blasts and the bone marrow microenvironment. Besides cellular elements of the microenvironment (e.g. mesenchymal stromal cells), bone marrow hypoxia has emerged as an additional crucial component. Hence, effects of hypoxia on FLT3 expression and biology could provide novel insight into AML biology. Here we show that 25% of AML patients down-regulate FLT3 expression on blasts in response to *in vitro* hypoxia (1% O_2_), which was independent of its mutational state. While virtually no AML cell lines regulate FLT3 in response to hypoxia, the down-regulation could be observed in Ba/F3 cells stably transfected with different FLT3 mutants. Hypoxia-mediated down-regulation was specific for FLT3, reversible and proteasome-dependent; with FLT3 half-life being significantly shorter at hypoxia. Also, PI-3K inhibition could partially abrogate down-regulation of FLT3. Hypoxia-mediated down-regulation of FLT3 conferred resistance against cytarabine *in vitro*. In conclusion, FLT3 expression in AML is dependent on the oxygen partial pressure, but response to hypoxia differs.

Acute myeloid leukaemia (AML) is a hematopoietic malignancy that develops from a single clone of a hematopoietic stem/progenitor cell. According to the two-hit hypothesis^1^,^2^, this leukaemia initiating cell (LIC) has acquired at least two driving mutations^3^. These mutations lead to (I) uncontrolled proliferation, (II) block in differentiation and (III) diminished apoptosis, resulting in the accumulation of leukemic blasts in the bone marrow. In recent years, an increasing number of mutations in AML have been identified and linked to differences in biology and prognosis. One of the most frequently mutated genes in AML is FLT3 (Fms-like tyrosine kinase^3^), a type III receptor tyrosine kinase with an important role in normal haematopoiesis^4^. It is expressed in early progenitors and the binding of its ligand (FLT3-L) triggers the activation of the PI3-K and the RAS pathways, sustaining proliferation and balance of apoptosis^5^. FLT3 is overexpressed in most haematological malignancies including 70% to 100% of AML^6^ and, if mutated, might contribute to leukaemogenesis by cooperation with other mutated molecules (K-ras, C-kit)^7^. The most common FLT3 mutation is the FLT3 internal tandem duplication (ITD), which occurs in 15–30% of AML patients and is thought to refer to a ligand-independent, constitutive activation, resulting in increased cell survival and proliferation of AML cells^4^. Presence of FLT3-ITD negatively impacts prognosis: although initial response rates are unaffected [CR]^8^, the risk of relapse is substantially higher^9^. Due to this prognostic impact and the possibility of pharmaceutical intervention by tyrosine kinase inhibitors (TKI) (e.g. sorafenib) therapeutic approaches to target wild type (WT) and mutated FLT3 have been extensively studied but have been by and large disappointing^10^. Interestingly, recent evidence suggests that the addition of sorafenib to chemotherapy improves event- and relapse- free survival but not overall survival in younger patients^11^. Relapse develops in the bone marrow where LIC are obviously able to survive anti-leukemic therapy^12^ by interacting with components of the bone marrow microenvironment^13^. As relapse rates in FLT3-ITD patients are higher than in FLT3-WT patients, one might assume a role for FLT3 in microenvironmental interactions, conferring e.g. survival advantage by rendering leukemic blasts less sensitive towards anti-leukemic drugs or TKI. The bone marrow microenvironment comprises cellular (e.g. perivascular and mesenchymal stromal cells)^14^ and non-cellular components (e. g. cyto- and chemokines)^14^, which usually work in concert for the maintenance of the normal haematopoiesis. Moreover, several studies have implied that the bone marrow is hypoxic, and characterised by heterogeneous areas of low oxygen levels that can vary from 6% O_2_ to 1% O_2_ 15,16. These low oxygen areas have been associated with physiological functions like differentiation of haematopoietic stem cells^17^. Hypoxia might influence AML development, and also alter biological features of AML like susceptibility towards chemotherapy^18^ and biology of cytokine receptor CXCR4^15^. CXCR4 is known to have a fundamental role in haematopoiesis (e.g. homing of haematopoietic stem cells towards the bone marrow niche) and it has additionally been linked to FLT3 in AML^19^. Hence, it seemed worthwhile to investigate the effects of hypoxia (as a physiological mainstay of the bone marrow microenvironment) on FLT3, in AML as well.

## Methods and Materials

### Patient samples

Primary samples were collected from AML patients who underwent routine diagnostic bone marrow aspiration or from peripheral blood, after informed consent was obtained. All were diagnosed with AML according to WHO criteria. Mononuclear cells were separated by Ficoll-Hypaque (Sigma-Aldrich, St Louis, MO, USA) density-gradient centrifugation and cultured at a density of 10^6^/ml in RPMI 1640 containing 10% FCS, 2 mM glutamine and 1% penicillin-streptomycin. All experiments were performed on fresh or frozen cells and approved by the local ethics committee (Ethic Committee of the University Hospital Großhadern, University of Munich) and carried out in accordance with the approved guidelines.

### Cells culture

All cell lines used (OCI-AML3, OCI-AML5, Mono-Mac-6, KG-1, Kasumi-1, NB-4, MV-4/11, Molm-13, HEL, CMK, M-07e, HL60,) were obtained from the DSMZ (German collection of Microorganisms and Cell Cultures, Braunschweig, Germany). Cell lines were seeded at a density of 2.5 × 10^5^/ml in RPMI 1640 containing 10% FCS (Biochrom, Berlin, Germany), 2 mM Glutamine and 1% penicillin-streptomycin (Life Technologies, Darmstadt, Germany). Cell numbers were evaluated by staining with trypan blue (Life Technologies, Darmstadt, Germany).

Ba/F3 cells transfected with different FLT3 mutants, receptor tyrosine kinases and cytokine receptors (Ba/F3 WT, Ba/F3 W51, Ba/F3 W78, Ba/F3 NPOS, Ba/F3 K644R, Ba/F3 EGF-R, Ba/F3 Epo-R) were seeded at a density of 5 × 10^4^/mL in growth medium with or without murine Interleukin (IL)-3 (5 ng/mL; Invitrogen, Camarillo, CA, USA). Standard laboratory (normoxic) conditions comprised 21% O_2_, 5% CO_2_, and 37 °C. For experiments in a reduced oxygen environment, the hypoxic Workstation INVIVO_2_ 400 from Ruskinn Technology (Bridgend, United Kingdom) was used, providing appropriate oxygen levels, 5% CO_2_, and 37 °C.

### Reagents

Cycloheximide (CHX) and proteasome inhibitor MG132 were purchased from Sigma-Aldrich (St Louis, MO, USA). PI-3K inhibitor LY294002 was purchased from Cell Signaling Technology (Danvers, MA, USA), HIF-hydroxylase inhibitor and Hsp90 inhibitor 17-AAG were purchased by Calbiochem (Darmstadt, Germany). Sorafenib was purchased from LTK Laboratories (St. Paul, MN, USA) and cytarabine from Sigma-Aldrich (St Louis, MO, USA).

### Western blotting

For Western Blot analysis, cells were lysed in 1× Cell lysis buffer (Cell Signaling Technology, Danvers, MA, USA) in either normoxia or hypoxia. Lysis buffer was supplemented with a protease inhibitor cocktail (Roche, Mannheim, Germany). Lysates were then separated on a 8% polyacrylamide gel, transferred to Hybond-P membranes (GE Healthcare, Little Chalfont, United Kingdom), probed with the appropriate antibodies FLT3 (c-20), EGF-R (1005), Epo-R (M-20) (all from Santa Cruz Biotechnology, Santa Cruz, CA, USA), c-KIT, p-STAT5, p-AKT (Ser473), GAPDH (all from Cell Signaling Technology, Danvers, MA, USA), β-actin (Sigma-Aldrich, St Louis, MO, USA) and visualized using an enhanced chemiluminescence kit (Thermo Scientific, Rockford, IL, USA). Relative quantification of protein bands from western blot films was performed by using ImageJ software and calculated as optical density (OD). OD was calculated as a ratio of each protein band relative to the lane’s loading control.

### Flow cytometry

Flow cytometric data were acquired using a FACSCalibur cytometer (BD Biosciences, San Jose, USA). Assessment of apoptotic cells were performed with the FITC Annexin V Apoptosis Detection Kit I (BD Pharmingen, Heidelberg, Germany). Analysis of Annexin-V expression was performed using BD CellQuest Pro software. After digest with 1 μg/ml RNAse A (Sigma-Aldrich, St Louis, MO, USA) cells were stained with 50 μg/ml PI (Sigma-Aldrich, St Louis, MO, USA) and DNA content was quantified by flow cytometry.

### Real-Time PCR

Total RNA was isolated from 10^6^ cells using MagNA Pure LC mRNA HS Kit (Roche, Basel, Switzerland) according to the manufacturer’s instructions. cDNA was synthesized from 1 μg aliquots of total RNA in a 20 μL standard reaction mixture using SuperScript® III First-Strand Synthesis System for RT-PCR (Invitrogen, Camarillo, CA, USA) according to the manufacturer’s instructions. Quantitative Real-time polymerase chain reaction (RT-PCR) was performed using the 7900HT Fast Real-Time PCR System (Applied Biosystems, Waltham, MA, USA) with 2 μL of cDNA, Fast SYBR® Green Master Mix (Applied Biosystems, Waltham, MA USA), and the following primers TGCCCGTCTGCCTGTAAAAT (forward) and AACCGGAATGCCAGGGTAAG (reverse) for FLT3. β-actin (Eurofins Scientific, Luxembourg, Luxembourg), was used as a reference for normalization of ΔCt values. All experiments were performed in triplicates.

### WST-Assay

Cytotoxycity was assessed by WST-1 assay according to the manufacturer’s instructions. After 48 hours 2.5 × 10^4^ Ba/F3 cells were exposed for 24 hours to increasing concentrations of cytarabine (Sigma-Aldrich, St Louis, MO, USA) or sorafenib (LTK Laboratories, St. Paul, MN, USA). 20 μl of WST-1 reagent were then added for a further incubation period of 3 hours. The ELISA reader (OptiMax, Molecular Devices, Sunnyvale, CA, USA) was set at a wavelength of 450 nm with a reference wavelength of 690 nm. Viability of cells after drug exposure was calculated as a percentage relative to untreated controls.

### CD34+ cell isolation

CD34+ cells were purified from not infiltrated bone marrow by magnetic bead separation using the human CD34 MicroBead kit and the MACS Pro separator (Miltenyi Biotec, Bergisch Gladbach, Germany) as instructed by the manufacturer. The purity of the CD34+ fraction was assessed by flow cytometry using an anti-CD34−PE antibody (Miltenyi Biotec, Bergisch Gladbach, Germany), and CD34+ fraction showing purity higher than 75% was used.

### Statistical analysis

Results are shown as the mean or median ± standard deviation of the mean (SEM) or standard deviation (SD) of at least 3 experiments each. Paired data were analysed using the paired Student *t* test. For survival analyses, the method of Kaplan-Meier using Log Rank test was used. Optical density (OD) was analysed using Image J Software. Microsoft Excel was used for data acquisition, storage and analysis, with additional calculations done with SPSS Statistics® (Version 22, IBM). A *P* value <.05 was considered statistically significant.

## Results

### FLT3 expression in AML subgroups is regulated by the oxygen partial pressure and might affect prognosis

**Figure 1:**
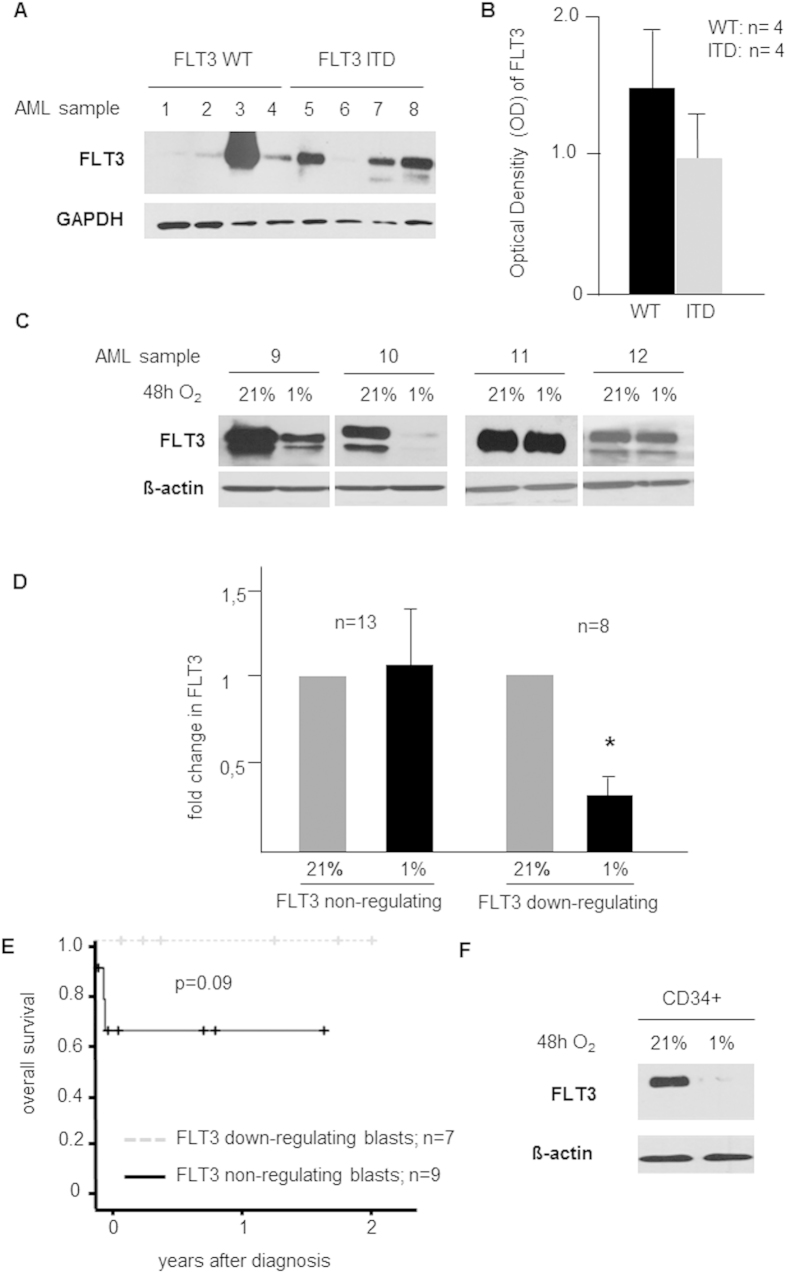
FLT3 expression in AML is regulated by the oxygen partial pressure. (**A**) Heterogeneous FLT3 basal expression in (n = 8; 4 WT, 4 ITD) AML patient samples was analyzed by western blot using whole cell lysates. (**B**) Expression of FLT3 was independent from its mutational state (optical density (OD) of FLT3 western blot bands normalized to GAPDH shown in Fig. 1; 4 WT, 4 ITD). (**C**) Western blot of representative examples (n = 4) of FLT3 down-regulating (sample 9 and 10) and non-regulating AML blasts (sample 11 and 12) after 48 h exposure to 1% O_2_. (**D**) Fold change of FLT3 OD normalized to β-actin. In FLT3 down-regulating AML cells (n = 8), down-regulation was 80% (*p* = 0.01), but in non-regulating cells (n = 13), FLT3 expression was virtually unchanged. (**E**) Clinical data available for n = 24 patients. Overall survival of patients without (n = 9) and with (n = 7) *in vitro* hypoxia-mediated FLT3 down-regulation (no detectable FLT3 in n = 8 patients). (**F**) Western blot analysis of whole cell lysates show down-regulation of FLT3 in healthy CD34+ cells in response to 1% O_2_.

In a first step, primary AML cells (n = 8) were analysed by western blot for their basal total FLT3 protein expression from uncultured cells. FLT3 expression levels were heterogeneous but independent from the mutational state of FLT3 (Fig. 1A,B). Next, we investigated by western blot analysis the effects of *in vitro* hypoxia of 1% O_2_ on FLT3 expression in AML patient samples (n = 33, 11 ITD, 22 WT). As expected, expression of FLT3 was highly variable: in 10 patients no FLT3 expression was observed (30%), while in 23 patients (70%), FLT3 levels could be detected. Surprisingly, in the latter group, we found two distinct behaviours concerning the effect of hypoxia on FLT3 expression (Fig. 1C): while 65% showed no difference in FLT3 expression after 48 hours of exposure to 1% O_2_ compared to 21%, in 35% of samples a significant down-regulation of FLT3 expression was observed (80% reduction at 1% O_2_, *p* = 0,01; Fig. 1D). The down-regulation of FLT3 was independent from the mutational state of FLT3 (down-regulators: ITD 57%, WT 42%, non-regulators: ITD 33%, WT 67%, not significant). Clinical data was available for 24 of these patients. Median age was 61.9 years [23.2–82.0] with 62.5% female patients. Seven samples showed *in-vitro* down-regulation of FLT3, 9 were non-regulators and in 8 patients no FLT3 was detected by western blotting. Adequate blast clearance (defined as <10% myeloblasts 7–10 days after completion of induction chemotherapy) was achieved in 100% of patients whose blasts showed hypoxia-mediated down-regulation of FLT3 *in* vitro and by 66% of not-regulating patients (not significant). Kaplan-Meier analysis revealed that patients with a regulating phenotype tended to live longer than patients without *in vitro* regulation of FLT3, however the difference was not significant (*p* = 0.09, Fig. 1E). The reason for the possible difference in survival was due to 3 early deaths, and interestingly, FLT3 non-regulators tended to have higher leukocyte count in the peripheral blood at the time point of initial diagnosis (107 G/l versus 35 G/l; *p* = 0.08). Additionally, FLT3 expression was analysed in normal CD34+ cells after 48 hours of exposure to 1% O_2_ and showed down-regulation of FLT3 as well (Fig. 1F).

### Hypoxia down-regulates FLT3 in the Ba/F3 cells stably transfected with FLT3

**Figure 2:**
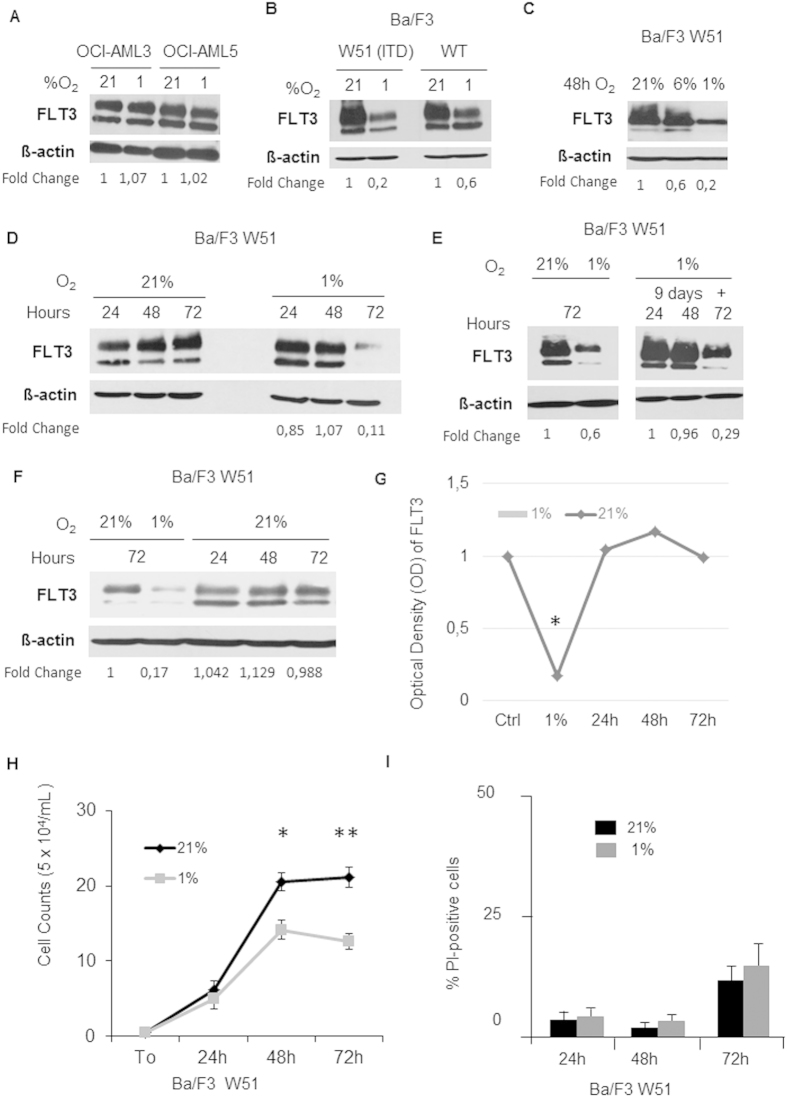
FLT3 is down-regulated by hypoxia in Ba/F3 cells. (**A**) OCI-AML3 and OCI-AML5 do not regulate FLT3 under hypoxia. Fold chance Optical Density (OD) was calculated compared to the respective value under 21% O_2_ and normalized to β-actin. (**B**) Ba/F3 cell lines stably transfected with FLT3 WT and FLT3 W51 (ITD) reliably down-regulate FLT3 at hypoxic conditions. (**C**) Down-regulation of FLT3 occurs in Ba/F3 W51 cells only below oxygen levels of 6% O_2_. (**D**) Ba/F3 W51 cells down-regulate FLT3 after 48 hours of culture under hypoxia. Fold change Optical Density (OD) was calculated for each time point compared to the respective value under 21% O_2_ and normalized to β-actin. (**E**) FLT3 down-regulation in Ba/F3 W51 cells is not a short-term stress hypoxic effect but is maintained for >9 days of culture under hypoxia. (**F**) Restoration of FLT3 expression after re-oxygenation of hypoxic Ba/F3 W51 cells with 21% O_2_. (**G**) Optical Density (OD) of FLT3 was calculated at different conditions compared to the respective value under 21% O_2_ at 72 hours and normalised to β-actin. (**H**) Significant decrease in proliferation in Ba/F3 W51 cells after 48 h and 72 h of culture at 1% O_2_. (**I**) No increased induction of cell death by hypoxia of 1% O_2_ in Ba/F3 W51 cells after 72 hours as compared to cells at 21% O_2_.

Next, we screened a panel of human leukemic AML cell lines (OCI-AML3, OCI-AML5, Mono-Mac-6, KG1, Kasumi-1, NB-4, MV-4/11, Molm-13, HEL, CMK, Mo7E, HL60) for FLT3 protein basal expression, which was not detectable in most of the cell lines. The cell lines with good detectable base line expression (Mono-Mac-6, OCI-AML3, OCI-AML5) were further investigated for FLT3 expression at 1% O_2_, however no FLT3 down-regulation was observed (two representative examples are shown in Fig. 2A). We than switched to Ba/F3 cells and analysed FLT3 expression in cells stably transfected with either FLT3-WT or FLT3-ITD construct W51. In this model, hypoxia induced down-regulation of FLT3 was reliably observed in all Ba/F3 cells independently from the mutational state of the receptor (Fig. 2B). As we were unable to detect significant differences between FLT3-WT and the FLT3-ITD mutant with respect to FLT3 down-regulation, Ba/F3 W51 (FLT3-ITD) cells were chosen for subsequent experiments due to their long-term IL-3 independent *in vitro* growth. With this *in vitro* model of hypoxia-mediated down-regulation of FLT3, we established the oxygen threshold required for down-regulation between 1% and 6% O_2_ (Fig. 2C) and the time window for down-regulation between 48 and 72 hours of hypoxia (Fig. 2D). FLT3 down-regulating phenotype was maintained after 9 and also after 12 days of hypoxia, suggesting that hypoxia-mediated FLT3 down-regulation is not a short-term stress reaction of AML cells towards 1% O_2_ (Fig. 2E). However, FLT3 down-regulation is reversible. Ba/F3 cells cultured for 3 days in hypoxic conditions showed a restoration of total protein within the first 24 hours after re-oxygenation with 21% O_2_ (Fig. 2F,G) and also exposure to fresh medium (data not shown). As expected, exposure to 1% O_2_ decreases proliferation of Ba/F3 cells (cell counts after 72 hours: 2.1 × 10^5^/ml for 21% O_2_, 1.3 × 10^5^/ml for 1% O_2_; *p* = 0.007, Fig. 2H), but viability was unaltered (88.7% viable cells at 21% O_2_ versus 85.4% at 1% O_2_ ; not significant, Figure I).

### Mechanism of FLT3 down-regulation

**Figure 3:**
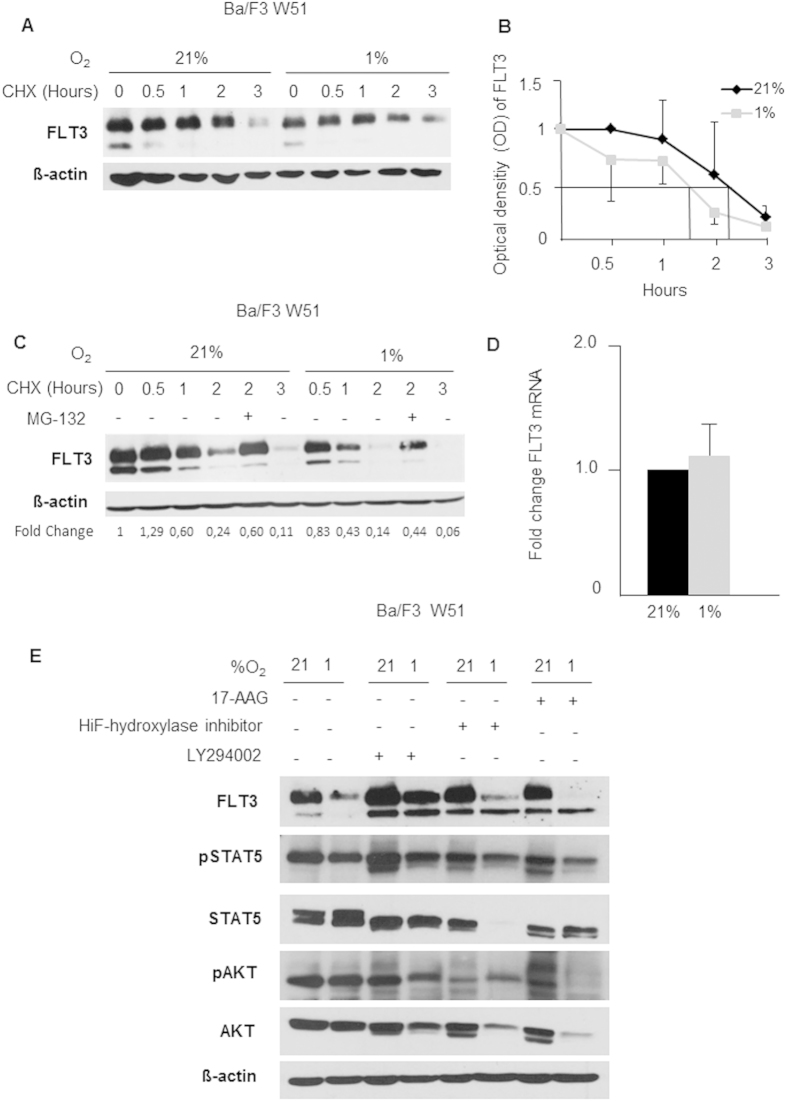
FLT3 regulating mechanisms by hypoxia. (**A**) Representative experiment of FLT3 degradation in Ba/F3 W51 at 21% and 1% O_2_ after protein synthesis inhibition with CHX (50 μg/mL) showing faster and increased degradation during hypoxia (immature intracellular FLT3 receptor: 130 kDa, mature glycosylated FLT3 receptor: 155 kDa). (**B**) Fold change of FLT3 OD normalized to β-actin. t½ is shortened during hypoxia by 1 hour from 2.5 hours to 1.5 hours. (**C**) Degradation of FLT3 in Ba/F3 W51 cells remains proteasome dependent also at 1% O_2_, as treatment with proteasome inhibitor MG132 reverses degradation after protein synthesis blockade with CHX. Fold change of FLT3 OD for each time point is calculated compared to the 0 time point value and normalized to β-actin (**D**) mRNA levels for FLT3 were unaltered after exposure to 72 hours of hypoxia in Ba/F3 cells. (**E**) Effects of HSP90 inhibition, Hif1alpha activation and PI3-Kinase inhibition on Ba/F3 W51 cells (cells were adjusted at hypoxia for 24 hours and treated for 48 hours) FLT3 expression and FLT3 downstream pathway activation was analysed by western blot. Only PI3-K inhibition partially abrogated hypoxia-mediated down-regulation of FLT3.

Half-life (t½) of FLT3 was reported as 2–3 hours^20^, however we found t½ of FLT3 at 1% O_2_ to be significantly shorter: as shown in Fig. 3A,B, degradation of FLT3 protein was fastened by 1 h at 1% O_2_, suggesting that hypoxia increases the turn-over of the protein. Treatment with the proteasome inhibitor MG132 (5 μM) reversed FLT3 degradation both at 1% O_2_ and at standard laboratory conditions (Fig. 3C), indicating that proteasome dependent degradation of FLT3 is also active during hypoxic conditions. Total levels of CBL were unaltered by hypoxia (data not shown). To investigate whether transcriptional mechanisms might also be involved in the different expression of FLT3 under hypoxic conditions, real-time PCR was performed for FLT3 mRNA levels at 1% O_2_ and 21% O_2,_ but no significant differences were observed in FLT3 transcript level at the different oxygen conditions (Fig. 3D). ELISA-based analysis of the supernatant of FLT3 down-regulating cells did not show detectable levels of FLT3, making extracellular shedding of the protein, as a potential mechanism of loss of FLT3 protein by hypoxia, highly unlikely (data not shown). To further identify possible other molecular mechanisms, we investigated the effects of phosphatidylinositide 3-kinase (PI3-K), Heat shock Protein 90 (Hsp90) inhibition and HIF-hydroxylase inhibitor (i.e. HIF1alpha stabilization) on hypoxia-mediated FLT3 down-regulation. As shown in Fig. 3E, FLT3 down-regulation could partially be abrogated by PI3-K inhibition but was neither affected by Hsp90 inhibition nor by HIF-1 alpha activation, suggesting that hypoxia-mediated FLT3-down-regulation might be partially regulated by the PI3-K pathway, but independent from HIF1alpha.

### Hypoxia mediated down-regulation is restricted to FLT3 protein, but independent from its mutational state

**Figure 4:**
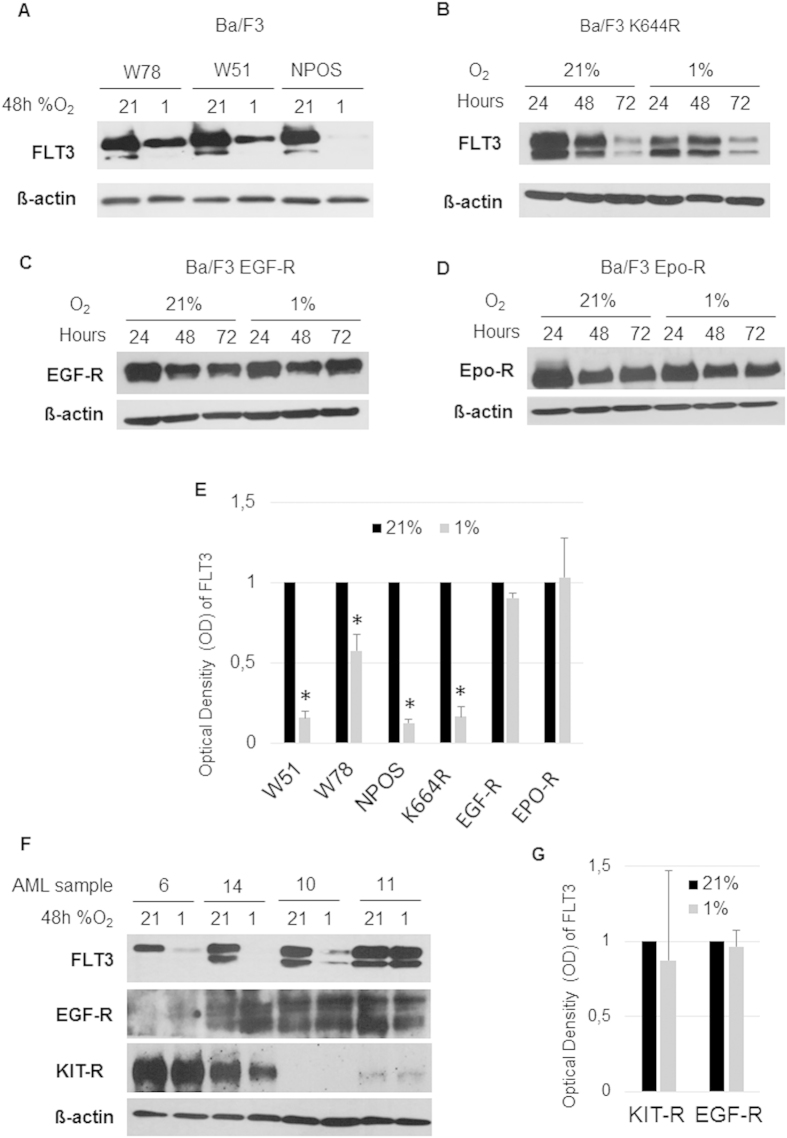
FLT3 down-regulation is a specific phenomenon. (**A**) Western blot of whole lysates of Ba/F3 cell line stably transfected with different FLT3 ITD constructs (W51, W78, NPOS) show down-regulation of FLT3 at hypoxic conditions. (**B**) Ba/F3 cells stably transfected with the kinase inactive FLT3 K644R mutant also down-regulated FLT3 under 1% O_2_. (**C**) No regulation of EGF-R by hypoxia of 1% O_2_ within 72 hours in Ba/F3 cells. (**D**) Ba/F3 cells stably transfected with Epo-R show no regulation of Epo-R by hypoxia. (**E**) Fold change of FLT3 OD normalized to β-actin in Ba/F3 W51, Ba/F3 W78 and Ba/F3 NPOS cells shows a statistical significance of FLT3 down-regulation under hypoxic conditions, whereas fold change of EGF-r and EPO-r OD do not show a significant down-regulation of this receptors under hypoxia. (**F**) No regulation of EGF-R and KIT receptor in n = 4 AML patient samples previously tested for FLT3 down-regulation under hypoxia (n = 3 down-regulating patients, n = 1 not regulating patient). (**G**) Optical density (OD) of KIT receptor and EGF-r western blot bands normalized to β-actin shows no statistical significance regulation under hypoxia in n = 4 AML patient samples.

Results in primary AML blasts and in Ba/F3 WT and W51 cells suggested that FLT3 down-regulation is independent from the mutational state of the receptor. To further validate this hypothesis, we investigated the expression of FLT3 at 1% O_2_ in 2 additional Ba/F3 FLT3-ITD mutants (Ba/F3 W78 and Ba/F3 NPOS) and in the kinase-inactive Ba/F3 K644R mutant. The hypoxia mediated down-regulation was also observed in each of these Ba/F3 cells (Fig. 4A,B). Next, we investigated whether hypoxia-mediated down-regulation is a general phenomenon applying to other tyrosine kinase receptors (TKR) and cytokine receptors as well; especially receptors that have a key role in haematopoiesis and leukaemogenesis as EGF-R, c-KIT and Epo-R. Ba/F3 cells stably transfected with EGF-R and Epo-R, were analysed for a 3-day time kinetic at 21% O_2_ and 1% O_2_ and, as shown in Fig. 4C,D, we failed to observe any hypoxia-induced regulation of the respective receptors.

All the Ba/F3 cells stably transfected with different form of FLT3 showed a significant down-regulation of the receptor under hypoxia compared to normoxia (Ba/F3 W51, p < 0,0001; Ba/F3 W78, p < 0,001; Ba/F3 NPOS, p < 0,0001; K644R p < 0,0001), whereas no significant regulation neither in EGF-r nor in Epo-R was observed under 1% O_2._

Expression levels of EGF-R and KIT receptor were checked in primary samples (n = 4) and results are shown in Fig. 4F: there was no regulation of EGF-R and KIT receptor under 1% O_2_, regardless of FLT3 regulation. In particular, individual differences between different patients were observed for KIT receptor expression under 1% O_2_ compared to 21% O_2_, nevertheless no significant down-regulation was observed in any of the patient sample tested (Fig. 4G).

### Functional consequences of FLT3 down-regulation

**Figure 5:**
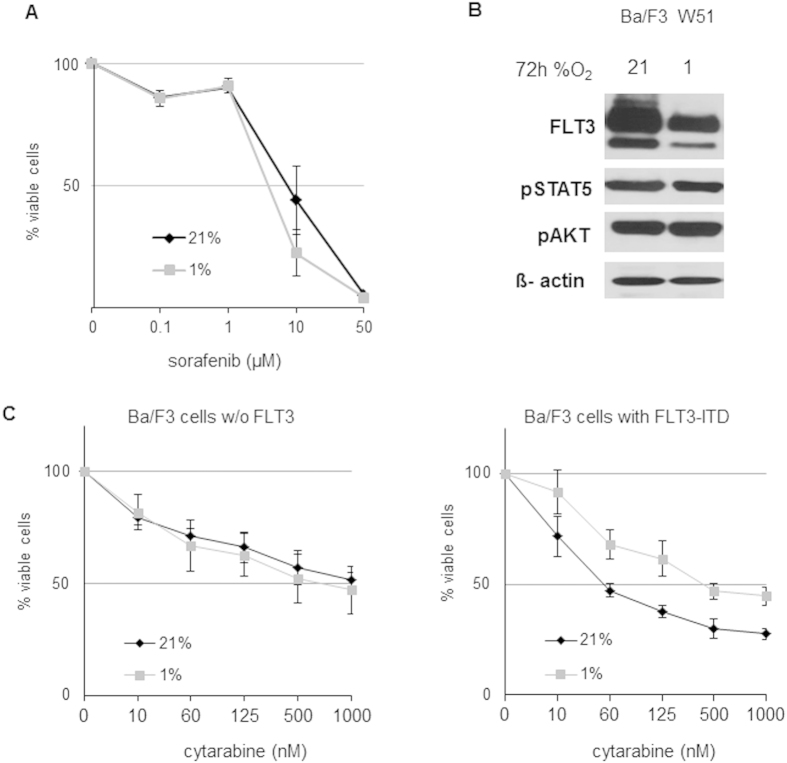
Functional consequences of FLT3 down-regulation. (**A**) Susceptibility of Ba/F3 W51 cells towards sorafenib (after 24 hours) was unaltered by the pO_2_. (**B**) No differences in STAT5 and AKT phosphorylation in Ba/F3 W51 cells cultured for 72 hours at 21% and 1% O_2_. (**C**) Ba/F3 cells transfected with an empty-vector and with Ba/F3 W51 cells were treated for 24 hours with increasing dose of cytarabine. FLT3-ITD affects susceptibility of Ba/F3 cells towards cytarabine at hypoxic conditions.

The further characterize functional consequences of FLT3 hypoxia-mediated down-regulation, we tested the susceptibility of Ba/F3 cells towards the tyrosine kinase inhibitor sorafenib, according to the expression level of FLT3 (i.e. 21% O_2_ versus 1% O_2_). Interestingly, no differences in the anti-leukemic efficacy of the drug were observed between hypoxia and normoxia (IC50 5.06 μM at 21%O_2_ versus 3.93 μM at 1% O_2_, not significant, Fig. 5A). In line with this finding was the observation that both canonical down-stream pathways of FLT3 (STAT5 and AKT) were unaltered by hypoxia as shown in Fig. 5B. However, FLT3 seems to be involved in the pharmacodynamics of cytarabine: while Ba/F3 cells transfected with an empty vector (negative control) where equally susceptible to cytarabine at normoxic and hypoxic conditions (IC50 287.17 nM versus 452.86 nM, not significant, Fig. 5C), the presence of FLT3 ITD in Ba/F3 W51 substantially reduced the susceptibility of these cells towards cytarabine at hypoxia. Results are shown in Fig. 5D: in contrast to normoxic conditions (n = 4; IC50 50.45 nM) the IC50 was not reached in all the experiments under hypoxic conditions (n = 3; IC50 413.88 nM).

## Discussion

FLT3 has been well studied in AML and mutations of this receptor belong to the oldest known genetic aberrations. The robust prognostic impact of FLT3 has been shown beyond doubt and a multitude of *in vitro* data has unravelled its biological function. However, therapies aimed at FLT3 have failed on various levels, and FLT3 targeted therapy has not entered standard therapy of AML. This suggests that large parts of FLT3 biology are still not understood. Several studies have implied in a direct or indirect way^16^,^21^,^22^, that the physiological state of the bone marrow is hypoxic and characterised by heterogeneous areas of low oxygen levels that can vary from 6% O_2_ to 1% O_2_. We have recently reported that normal and leukemic bone marrow do not differ in their low oxygen levels^23^, suggesting that the definition of hypoxia in the context of AML differs from the ones used in other malignancies, where it is used to describe a non-physiologic condition, e.g. a non-physiologic hypoxic or anoxic core arising from insufficient neo-angiogenesis. The data presented here demonstrate that the oxygen partial pressure (pO_2_), significantly influences expression and biology of FLT3. Hypoxia of 1% O_2_ but not 6% O_2_ down-regulates FLT3 expression in (I) appr. 25% of AML patients, (II) in normal CD34+ haematopoietic progenitors and (III) in the Ba/F3 cell line stably transfected with different FLT3 constructs. Down-regulation of FLT3 by hypoxia was irrespective whether the protein was mutated or not and of the carried mutation. In contrast, hypoxia-induced down-regulation of FLT3 does not occur in appr. 75% of AML patients and in virtually none of the investigated AML cell lines. The question that arises from this finding is whether down-regulation of FLT3 is a normal physiological consequence of hypoxia. Concluding from our data, we would support this hypothesis: (I) normal CD34+ positive cells showed FLT3 down-regulation, (II) the down-regulation was “specific” to FLT3 and not observed in other TKR and cytokine receptor relevant in haematopoiesis and (III) AML patients with a down-regulating phenotype tend to have a better prognosis (longer survival, higher CR rates and lower peripheral blast counts). This hypothesis is supported by the observation that in AML, high FLT3 expression levels confers a tendency towards worse survival^24^. Moreover the finding that 12 AML cell lines studied (which are usually established from more “aggressive” types of leukaemia with autonomous *in vitro* growth) do not regulate FLT3, further suggest that the non-regulating phenotype is not physiological, and that some AML blasts lose the property of a physiological, hypoxia-mediated FLT3 regulation. We chose to further characterize the phenomenon of hypoxia-mediated down-regulation of FLT3 – but admittedly the identification of loss of this feature could be rewarding in the context of AML as well. As only primary AML samples and FLT3 transfected Ba/F3 cells showed the hypoxia-mediated down-regulating phenotype, the latter murine system was chosen as a model for further experiments. It was found that the down-regulation of FLT3 by hypoxia follows the “normoxic” conditions, as the down-regulation remained proteasome dependent but with a t½ that was 1 hour shorter at hypoxia of 1% O_2_. Transcriptional mechanisms seemed not to be involved in FLT3 down-regulation. Interestingly, PI3-K might act as a possible relevant pathway connected to hypoxia-mediated FLT3 down-regulation. PI3-K pathway is one of the most frequently activated in AML and also activated by hypoxia, potentially by increased lipid raft formation^22^. Additionally, binding of FLT3 ligand to FLT3 activates PI3-K^6^, whereas mutated FLT3 signals through STAT5 and JAK pathways^25^,^26^. Surprisingly, in the murine system, none of these latter pathways were altered in every aspect we investigated, neither by hypoxia nor by the expression levels of FLT3. By inhibiting PI3-K pathway we could partially reverse the FLT3 down-regulation, indicative for a negative feedback loop between FLT3 and PI3-K at hypoxic conditions. The question how AML blasts and cell lines lose their ability to regulate FLT3 in response to pO_2_ remains elusive. A “simple” mutation in a protein involved in FLT3 metabolism (e.g. in degradation as CBL^27^ seems unlikely in the light of the fact that the un-regulating phenotype was observed in 75% of patients. A mutation with that frequency would have certainly been already detected. Hence, other mechanisms like different expression of degrading proteins or epigenetic mechanisms could be responsible. The effects however may be more obvious; increased expression of FLT3 is known to be associated with an inferior prognosis^24^, and presumably the increased expression of FLT3 receptor might result in increased FLT3-L binding and hence increased FLT3 signalling and activation of PI3-K – comparable to a constitutive activation. Hence, a constitutive expression of that receptor that eludes physiological regulation might result in the escape from environmental control. However, FLT3 might have other, hitherto unknown functions as well: surprisingly, we found that FLT3 itself affected the susceptibility of cells to cytarabine, as cells lacking FLT3 showed no difference in response to cytarabine according to oxygen in contrast to cells expressing FLT3. Whether and how FLT3 is involved in the pharmacodynamics of cytarabine certainly deserves further investigations, given the importance of cytarabine in the treatment of AML. In conclusion, these data show that FLT3 is regulated in hematopoietic cells by the oxygen partial pressure (as observed in the hematopoietic stem cell niche in the bone marrow) and that a substantial proportion of AML cells lose this phenotype. Whether this is an AML initiating event or a side effect of the e.g. well established hematopoietic differentiation block in AML needs to be further investigated, as well as the functional consequences.

## Additional Information

**How to cite this article**: Sironi, S. *et al.* Microenvironmental Hypoxia regulates FLT3 expression and biology in AML. *Sci. Rep.* **5**, 17550; doi: 10.1038/srep17550 (2015).
